# Lactose Glycation of the Maillard-Type Impairs the Benefits of Caseinate Digest to the Weaned Rats for Intestinal Morphology and Serum Biochemistry

**DOI:** 10.3390/foods10092104

**Published:** 2021-09-06

**Authors:** Xiao-Peng Wang, Xin-Huai Zhao

**Affiliations:** 1 College of Food Science and Technology, Henan Agricultural University, Zhengzhou 450002, China; hnndwxp@henau.edu.cn; 2 Key Laboratory of Dairy Science, Ministry of Education, Northeast Agricultural University, Harbin 150030, China; 3 School of Biology and Food Engineering, Guangdong University of Petrochemical Technology, Maoming 525000, China; 4 Research Centre of Food Nutrition and Human Healthcare, Guangdong University of Petrochemical Technology, Maoming 525000, China; 5 Maoming Branch, Guangdong Laboratory for Lingnan Modern Agriculture, Guangdong University of Petrochemical Technology, Maoming 525000, China

**Keywords:** glycated caseinate, Maillard reaction, weaned rat, growth performance, intestinal morphology, digestive enzyme, brush-border enzyme, serum biochemistry

## Abstract

The Maillard reaction between the lactose and milk proteins unavoidably occurs during the thermal treatment of milk. Although the impact of this reaction on protein nutrition and safety has been well-studied, whether a lactose glycation of milk proteins of the Maillard-type might affect the rats in their growth and intestinal morphology needs an investigation. In this study, caseinate and lactose-glycated caseinate were digested using pepsin and trypsin. Afterward, the resultant caseinate digest and glycated caseinate digest (lactose content of 13.5 g/kg of protein) at 100, 200, and 400 mg/kg body weight (BW)/d were assessed for their effects on the female weaned Wistar rats in terms of daily body weight gain, intestinal morphology, digestive and brush-border enzyme activities, as well as serum chemical indices. The results showed that glycated caseinate digest always showed a weaker effect on rat than caseinate digest either at the 0–7 or 0–28 d feeding stage, and more importantly, at the highest dose of 400 mg/kg BW/d, it caused obvious adverse effect on the rats, reflected by lower values of these indices. Compared with caseinate digest, glycated caseinate digest in the rats caused 0.9–15.4% and 10.6–49.7% decreases in average daily gain of BW and small intestinal length, 1.1–21.5% and 2.3–33.3% decreases in villus height and the ratio of villus height to crypt depth of the small intestine, or 0.3–57.6% and 0.2–55.7% decreases in digestive and critical brush-border enzyme activities, respectively. In addition, when the rats were fed with glycated caseinate digest, some serum indices related to oxidative stress status were enhanced dose-dependently. Lactose glycation of the Maillard-type is thus considered as a negative event of the Maillard reaction on milk proteins because this reaction might impair protein benefits to the body.

## 1. Introduction

Dairy products such as the ultra-high temperature (UHT) milk, milk powders, and infant formulas are always subjected to considerable heat treatment, which are essential for the microbiological safety and shelf life [1]. However, heat treatment that is especially carried out at higher temperature may decrease the nutritive values of dairy products and generate various Maillard products that might be detrimental to human health. Major changes in dairy products caused by heat treatment are largely or partly due to the Maillard reaction, which is unavoidable because of the existence of both reducing sugar lactose and milk proteins [2]. The Maillard reaction principally occurs between lactose and the lysine residues of caseins, as these lysine residues are more reactive than those from whey proteins [3]. In general, this reaction can be divided into three (i.e., early, middle, and terminal) steps, while the reaction extent depends on both thermal procedures and storage conditions [4]. The most dominant product of this reaction is the lactulosyl-lysine, an Amadori product formed at the early reaction stage [5]. When severe heat treatment is used in dairy processing, the Amadori product will be converted into further reaction products, the advanced glycation end products (AGEs) [6]. It was evident that proteins modified via Maillard reaction had enhanced functional properties and bioactivities, including solubility, emulsification, anti-oxidation, and anti-bacterial effect [7,8]. However, this reaction in milk or dairy products can cause several events including brown discoloration, pH reduction, production of flavor compounds, and decreased nutritive values [9]. Most significantly, AGEs can enter the body and thus possess an ability to affect body health. It was found that, after one week of daily feeding with the heated or untreated skimmed milk powder, 25 metabolites were detected in the urine samples of the rats, while 19 metabolites were regarded as the lysine/arginine-derived AGEs and heterocyclic compounds [10]; moreover, AGEs can promote oxidative stress and inflammation, alter glucose regulation, and increase the formation of endogenous AGEs [11,12]. A growing body of evidence also has shown the role of non-enzymatic protein glycation in the development of diabetic vascular complications including the diabetic nephropathy, retinopathy, and chronic kidney disease [13,14]. In addition, the potential impact of this glycation might be more significant when dairy products are the main source of essential amino acids, such as the infant diet. Thus, protein modification including glycation in infant formulas was proposed leading to health risk for the infants consistently exposed to high levels of infant formulas [15]. From a chemical point of view, the glycated proteins are the products of early stage of this reaction, but have chemical structures different from their parent proteins (i.e., conjugated saccharide molecules). Thus, the possible effect of the resultant glycated proteins on human health or body development requires further investigation.

The intestine is an important internal environment in the body where a number of processes occur in order to nourish the body and to protect it against the invasion of harmful substances [16]. The intake of various substances containing in the daily diets is thought to impact intestinal function [17]. Unlike substances such as polyphenols and the indigestible oligosaccharides, most proteins are hydrolyzed into peptides at the intestinal tract and epithelium [18]; thus, some peptides with potential bioactivity to the intestine are produced. The lactose-glycated caseins exist in processed dairy products due to the mentioned Maillard reaction, while intake of these glycated proteins into the gastrointestinal tract will generate respective glycated digests via the action of digestive proteases. Compared with the unglycated digests, whether the glycated digests have increased or decreased bioactivity to the body is absolutely important. Two previous studies by our group had found that the lactose-glycated caseinate digest had poor ability compared with the unglycated caseinate digest to promote barrier integrity of rat intestinal epithelial (IEC-6) cells or to combat against the camptothecin-induced apoptosis in IEC-6 cells [19,20]. Whether the lactose-glycated digest has a changed in vivo activity toward the weaned animals than the unglycated digest, for example, the vital body growth and intestinal development, is not investigated yet. Such a study thus attracts our attention. To provide extra evidence for the conclusion from the two previous studies [19,20], this study also used the two digests in the animal experiment.

In this study, a lactose-glycated caseinate digest was thus prepared using the Maillard reaction of lactose and caseinate followed by a digestion with two digestive proteases (pepsin and trypsin). Using caseinate digest as a control, the obtained glycated caseinate digest was evaluated for its effect on body weight gain and serum biochemical indices of the weaned rats at two development stages (0–7 and 0–28 d). Special attention was also given to intestinal development via assaying the changes of intestinal morphology as well as digestive and critical brush-border enzyme activities. The aim of this study was to explore a possible in vivo risk of the Maillard reaction products that might be generated during the thermal processing of dairy products.

## 2. Materials and Methods

### 2.1. Chemicals and Reagents

The caseinate (protein content of 984.2 g/kg on dry basis) and D-lactose were purchased from Sigma-Aldrich Co., Ltd. (St. Louis, MO, USA). Both pepsin and trypsin (measured respective activities of 40 and 120 U/mg) were provided by Beijing Aoboxing Biotechnologies Inc. (Beijing, China). The galactose assay kit was purchased from BioAssay Systems (Hayward, CA, USA). The alkaline phosphatase and bicinchoninic acid protein assay kits were bought from Beyotime Institute of Biotechnology (Shanghai, China). The lipase and α-amylase assay kits were bought from Nanjing Jiancheng Bioengineering Institute (Nanjing, Jiangsu, China). In addition, the water used in this study was ultrapure water generated by Milli-Q Plus (Millipore Corporation, New York, NY, USA). Other chemicals used in this study were of analytical grade.

### 2.2. Preparation and Chemical Analyses of the Digests

The preparation and enzymatic digestion of the lactose-glycated caseinate was performed as previously described [21]. Briefly, the reaction mixture was prepared with caseinate (50 g/L) and lactose (80 g/L) at pH 6.8, followed by a reaction at 100 °C for 3 h. After an isoelectric precipitation and water washing (three times), the collected precipitate (i.e., lactose-glycated caseinate) was freeze-dried. Subsequently, the digestion of the glycated caseinate was carried out by pepsin (800 U/g protein) for 1 h at 37 °C, followed by tryptic digestion (7 kU/g protein) at pH 7.0 and 37 °C for 2 h. The unglycated caseinate was also digested as the glycated caseinate. The yielded caseinate (CN) digest and glycated caseinate (GCN) digest were then stored at −20 °C before their use in chemical or cell experiments.

Protein content was assessed using the Kjeldahl method and a conversion factor of 6.38 [22]. To assay lactose content of the glycated digest, the sample was hydrolyzed by 2 mol/L trifluoroacetic acid at 100 °C for 4 h, cooled to 20 °C, and neutralized with 0.5 mol/L NaOH solution. The obtained hydrolysate was detected for galactose content and then calculated for lactose content (g/kg protein), as previously described [19].

### 2.3. Animals, Diets, and Housing

Ninety female Specific Pathogen-Free (SPF) weanling Wistar rats weighing 40.26 ± 0.58 g were provided by Beijing Vital River Experimental Animal Technical Co. Ltd. (Beijing, China) (qualification certificate number: 11400700220420). All animal procedures were performed and supervised in compliance with the guidelines of the Animal Ethics Committee of Northeast Agricultural University (Harbin, Heilongjiang, China) under the approved protocol number of NEAUEC20190316. The rats were housed in the steel cages (three rats in one cage) under standard conditions (specific pathogen free, temperature 20–22 °C with a 12 h light/dark cycle and 55–57% humidity), and had ad libitum access to their diets and de-mineralized water.

Six rats were sacrificed to obtain the initial index values. The other rats were average divided into seven groups and randomly assigned to one of the mentioned treatments. One group of the rats was designed as control and treated daily by gavage with physiological saline solution (0.9% NaCl). Three groups of the rats were given CN digest daily of three dose levels by the intra-gastric administration, while the other three groups of the rats were given GCN digest of the same doses. The used doses were 100, 200, and 400 mg/kg BW/d, based on the reported experimental indices [23,24]. Each group was then evenly divided into two parts. Six rats were fed for 7 d to observe the effect of the digests on the infant stage of the rats, while the other six rats were fed for 28 d to analyze the impact of the digests on the adult rats. Rat BW was monitored daily throughout the whole experimental period; moreover, the rats were fasted for 6 h except for water prior to the sacrifice. The lengths and weights of the small intestine of the rats were measured after being anesthetized and sacrificed on the expiration of gastric perfusion. The length of the small intestine was designated from the pylorus sphincter to proximal of the ileocecal valve. Similarly, the duodenum (from the gizzard to bile duct), jejunum (from the bile duct to Meckel’s diverticulum), and ileum (from the Meckel’s diverticulum to ileocecal junction) were segmented.

### 2.4. Assay of Intestinal Morphology

All rats were sacrificed under anesthesia by intraperitoneal injection of chloral hydrate solution (250 mg/kg). Based on the anatomical hallmarks, the samples of 1 cm length were harvested from the duodenum, jejunum, and ileum, respectively. The tissue specimens were all fixed in 10% neutral buffered formalin and dehydrated for 12 h. On the following day, the specimens were cut with a razor blade and then stored in 70% ethanol for paraffin embedding. Five-micron tissue slices were stained with the hematoxylin-eosin (H&E) staining. Crypt depth and villus height were observed from the H&E stained slides under a light microscope equipped with a digital camera, and measured with Image J Computer Software version 1.46 (Bethesda, MD, USA).

### 2.5. Assays of Digestive and Brush-Border Enzyme Activities

The mucosa (for brush-border enzyme activities) and chyme (for digestive enzyme activities) samples from the duodenum, jejunum, and ileum were individually thawed and homogenized in 4 volumes of the ice-cold 0.9% NaCl. The homogenates were sonicated 10 times for 6 s each in ice bathing and centrifuged for 5 min (12,000× *g*, 4 °C) to obtain the supernatants that were used for the assays as below. Protein concentrations of the supernatants were assessed using the bicinchoninic acid protein assay kit and protocol provided by kit manufacturer.

The lipase (EC 3.l.l.3) activity was assayed using a colorimetric method at 420 nm, as recommended by the kit manufacture. Lipase activity unit was defined as a hydrolysis of 1 μmol triglycerides in 1 min at 37 °C and expressed as units (U)/g protein. In addition, the α-amylase (EC 3.2.1.1) activity (U/g protein) was assayed using the colorimetric method at 660 nm according to the kit instruction, defined as a hydrolysis of 10 mg starch in 30 min at 37 °C. Trypsin (EC 3.4.21.4) was measured using caseinate as the substrate, while the enzymes activity was expressed as U/g protein. One unit of trypsin activity was defined as the formation of 1 μg tyrosine per minute at 37 °C [25].

Lactase (EC 3.2.1.22) and sucrase (EC 3.2.1.26) activities were measured as previously described [19]. Briefly, the supernatants were incubated at 37 °C with 50 mmol/L lactose and sucrose for 60 and 30 min, respectively. The liberated glucose was measured using the glucose assay kit. Lactase (EC 3.2.1.22) and sucrase (EC 3.2.1.26) activities were expressed as nmol glucose/mg protein/min [26]. Alkaline phosphatase (EC 3.1.3.1) activity (U/mg protein) was measured using para-nitrophenyl phosphate as substrate [27], while the liberated p-nitrophenol was measured at 405 nm using the alkaline phosphatase assay kit.

### 2.6. Assays of Serum Chemical Indices

Serum chemical assay was performed at Heilongjiang Electric Power Hospital (Harbin, Heilongjiang, China). In brief, 24 h after the last administration, the blood samples from the rats were collected using the abdominal veins collection. The serum samples were separated by a centrifugation at 2000× *g* for 10 min, and then analyzed at the Beckman DXC 800 Automatic Chemistry Analyzer (Beckman Coulter, Inc., Brea, CA, USA) to obtain the values of low-density lipoprotein, glucose, triglyceride, cholesterol, blood urea nitrogen, alanine transaminase, aspartate amino transferase, and other indices, as previously described [28,29].

### 2.7. Statistical Analysis

All data were expressed as means ± standard deviations by evaluating all rats with at least six independent assays. The results were submitted to Shapiro–Wilk tests for normal distribution and then examined using one-way analysis of variance (ANOVA) with Duncan’s multiple range test. Paired samples *t* test was used to determine data differences between the rats fed for 7 and 28 d, while correlation analysis was performed using the Pearson’s correlation test. The statistical significance was set at a level of *p* < 0.05. The SPSS software version 16.0 (SPSS Inc., Chicago, IL, USA) and SigmaPlot version 12.5 (Systat Software Inc., San Jose, CA, USA) were used in data analysis and figure plotting, respectively.

## 3. Results

### 3.1. The Body Weight Gain and Intestinal Indices of the Rats in Response to the Two Digests

**Figure 1:**
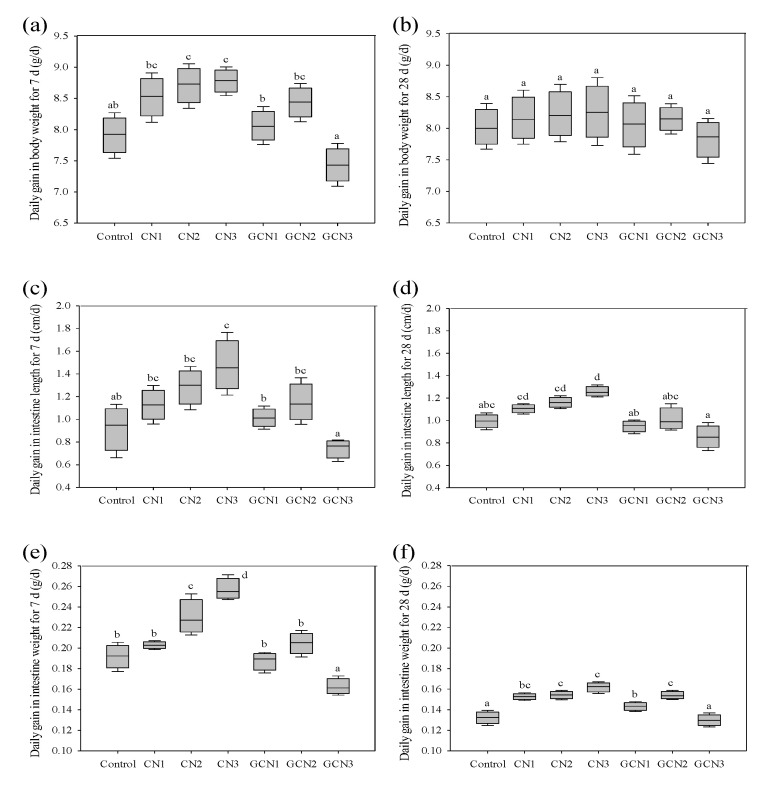
Effects of caseinate digest and glycated caseinate digest on daily gain in BW for 7 or 28 d (**a**,**b**), intestine length for 7 or 28 d (**c**,**d**), and intestine weight for 7 or 28 d (**e**,**f**). Values are reported as means ± standard deviations (*n* = 6), while different lowercase letters above the columns indicate that one-way ANOVA of the means differs significantly (*p* < 0.05). CN: caseinate; GCN: glycated caseinate; control: rats fed with 0.9% NaCl; CN1, CN2, and CN3: rats fed with 100, 200, and 400 mg/kg BW/d of CN digest, respectively; GCN1, GCN2, and GCN3: rats fed with 100, 200, and 400 mg/kg BW/d of GCN digest, respectively.

In this study, the prepared glycated caseinate contained lactose of 14.29 g/kg protein, while the obtained GCN digest contained lactose of 13.51 g/kg protein. However, CN digest did not have detectable lactose. During the whole experimental period, all rats in the seven groups survived. At the 7- or 28-day experimental stages, the changes of three indices including average BW, small intestine weight, and small intestine length were assessed to reflect rat growth in response to the intake of CN digest and GCN digest. The obtained data are partly given in Figure 1 and the Supplementary Materials (Table S1).

In total, the two digests dose-dependently (except for GCN at 400 mg/kg BW/d) had an enhancing effect on the rats (*p* < 0.05), mostly by causing value increases for these indices; however, GCN digest at 400 mg/kg BW/d had a negative effect on the rats, because it caused the lowest values for these indices. In detail, when the rats were fed for 7 d, CN digest and GCN digest caused 8.52–8.78 g and 7.43–8.44 g daily gain in average BW, respectively. Compared with CN digest, GCN digest resulted in 3.1–15.4% value decreases for this index. At the same time, CN digest resulted in average small intestine length of 1.13–1.47 cm and average small intestine weight of 0.20–0.26 g, while GCN digest led to index values of 0.74–1.15 cm and 0.16–0.20 g, respectively. Regarding CN digest, GCN digest yielded 10.6–49.7% and 5.0–38.5% value decreases for the two indices. When the rats were fed for 28 d, CN digest and GCN digest induced 8.16–8.26 g and 7.83–8.15 g daily gain in average BW, respectively. At the same time, CN digest caused index values of 1.11–1.26 cm and 0.15–0.16 g for the average small intestine length and weight, while GCN digest yielded the lower values of 0.85–0.97 cm and 0.13–0.15 g for the two indices, respectively. Clearly, GCN digest in the rats always brought about lower index values than CN digest at the same dose level. These results thus suggested that the caseinate lactose-glycation of the Maillard-type had an unfavorable effect on the bioactivity of GCN digest in the rats.

### 3.2. The Morphology Changes of Intestinal Tissues in Response to the Two Digests

**Figure 2:**
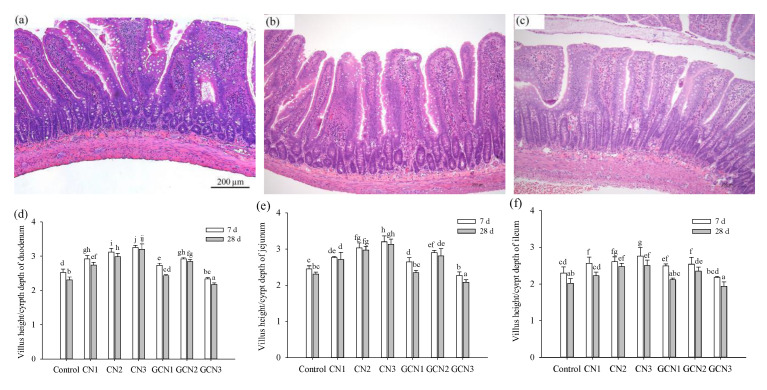
The histological pictures (**a**–**c**) and calculated villus height/crypt depth (**d**–**f**) of the duodenum specimens obtained from the control rats (**a**,**d**), the rat fed with CN digest (**b**,**e**) or GCN digest (**c**,**f**) at dose level of 400 mg/kg BW/d. Values are reported as means ± standard deviations (*n* = 6), while different lowercase letters above the columns indicate that one-way ANOVA of the means differs significantly (*p* < 0.05). CN: caseinate; GCN: glycated caseinate; control: rats fed with 0.9% NaCl; CN1, CN2, and CN3: rats fed with 100, 200, and 400 mg/kg BW/d of CN digest, respectively; GCN1, GCN2, and GCN3: rats fed with 100, 200, and 400 mg/kg BW/d of GCN digest, respectively.

The morphology changes of the rat intestinal tissues are shown in the Figure 2 and the Supplementary Materials (Table S2). Briefly, the two digests in dose-dependent manner enhanced the values of villus height (VH) and the ratio of VH to crypt depth (VH/CD) for the duodenum, jejunum, and ileum (except for using GCN digest of 400 mg/kg BW/d). Notably, GCN digest of 400 mg/kg BW/d brought about an adverse effect on the rats, because the rats thus fed were measured with the lowest values for the two indices. Compared with the control rats fed for 7 d, the rats fed with CN digest at 100–200 mg/kg BW/d for 7 d had increased VH and VH/CD values of 9.6–17.8% and 12.6–23.8%, while those fed with GCN digest at 100–200 mg/kg BW/d for 7 d had enhanced VH and VH/CD values of 1.5–12.2% and 7.8–18.4%, respectively. GCN digest thus showed 2.3–8.0% lower efficacy than CN digest to increase these index values. At the same time, regarding the control rats fed for 28 d, the rats fed with CN digest at 100–200 mg/kg BW/d for 28 d had increased VH and VH/CD values of 6.1‒11.0% and 10.4–29.6%, while those fed with GCN digest at 100–200 mg/kg BW/d for 7 d only had enhanced VH and VH/CD values of 1.3–8.3% and 1.7–23.5%, respectively. GCN digest also showed lower efficacy (1.1–13.0%) than CN digest in this case. Thus, the performed lactose-glycation of caseinate is proposed to suppress the benefit of GCN digest to enhance VH and VH/CD values.

### 3.3. The Changes of Digestive and Brush-Border Enzyme Activities in Response to the Two Digests

The measured activities of the three digestive enzymes (lipase, α-amylase, and trypsin) in the duodenum, jejunum, and ileum are listed in Table 1, which shows that the two digests mostly could enhance the activities of these enzymes (except for using GCN digest of 400 mg/kg BW/d). Compared with the control rats fed for 7 d, the rats fed with CN digest at 100–200 mg/kg BW/d had respective 5.4–55.5%, 18.4–38.7%, and 29.1–88.5% value increases in lipase, α-amylase, and trypsin, while those fed with GCN digest at 100–200 mg/kg BW/d for 7 d had enhanced values of 0.7–43.8%, 4.7–38.2%, and 2.5–56.0% in the three enzymes, respectively. GCN digest was thus less efficient (0.3–25.6%) than CN digest to increase these index values. When the rats were fed for 28 d, the rats fed with CN digest 100–200 mg/kg BW/d had increased lipase, α-amylase, and trypsin activities by 8.6–27.4%, 8.5–48.3%, and 42.7–73.3%, while those fed with GCN digest at the same doses for 28 d had enhanced values of 1.2–25.8%, 5.6–45.8%, and 0.4–50.6% for the three enzymes, respectively. GCN digest thus showed 0.3–29.6% lower efficacy than CN digest in this case. In addition, it was also evident that feeding time of 7 or 28 d for the rats led to obvious activity differences for the three enzymes (*p* < 0.05), except for the ileum trypsin from the rats fed with GCN digest of 200 mg/kg BW/d. Moreover, correlation analysis results also indicated that the daily BW gain was not correlated with digestive enzyme activities (*p* > 0.05).

The detected activities of the three critical brush-border enzymes (lactase, sucrase, and alkaline phosphatase) in the duodenum, jejunum, and ileum are given in Table 2, in which GCN digest at 400 mg/kg BW/d led to decreased enzyme activities. In detail, compared with the control rats fed for 7 d, the rats fed with CN digest at 100–200 mg/kg BW/d for 7 d had respective 6.4–27.3%, 20.0–61.6%, and 18.1–57.5% value increases for lactase, sucrase, and alkaline phosphatase activities, while those fed with GCN digest at 100–200 mg/kg BW/d for 7 d had enhanced activity values of 5.0–14.1%, 2.4–43.2%, and 6.4–31.1%, respectively. CN digest thus showed 1.3–22.4% higher capacity than CN digest to increase these enzyme activities. When the rats were fed for 28 d, the rats fed with CN digest at 100–200 mg/kg BW/d for 28 d had increased lactase, sucrase, and alkaline phosphatase activities by 10.6–22.3%, 23.4–61.3%, and 5.3–40.4%, while those fed with GCN digest at 100–200 mg/kg BW/d for 28 d had enhanced activity values of 1.0–21.3%, 7.3–53.9%, and 2.9–16.9%, respectively. In this case, CN digest was also more efficient (0.8–24.2%) than GCN digest in the rats. Moreover, feeding time of 7 and 28 d also led to obvious activity differences for the three brush-border enzymes (*p* < 0.05), except for the duodenum lactase and alkaline phosphatase from the rats fed with CN digest of 200 and 400 mg/kg BW/d. However, the results from correlation analysis also indicated that there was no obvious correlation (*p* > 0.05) between daily BW gain and the brush-border enzyme activities.

Based on the findings that GCN digest at 400 mg/kg BW/d clearly decreased these enzyme activities while, at 100–200 mg/kg BW/d, it was less efficient than CN digest to enhance these enzyme activities, it is regarded that the lactose-glycation of caseinate caused an adverse effect on GCN digest for its ability to enhance the activities of these assessed digestive or brush-border enzymes.

### 3.4. Serum Biochemical Indices of the Rats in Response to the Two Digests

The results (Table 3) illustrate the effects of CN digest and GCN digest on the 7 important blood biochemical indices of the rats, while the data given in the Supplementary Materials (Table S3) show the value changes of other serum biochemical indices of the rats. Compared to the control rats, the rats fed with CN digest at lower doses (100 and 200 mg/kg BW/d) were observed with insignificant value changes either in the experimental period of 7 or 28 d, while the highest dose of CN digest (400 mg/kg BW/d) only caused 4.6–17.4% value increases for the 7 indices (Table 3). Conversely, compared with the control rats fed for 7 d, the rats fed with GCN digest for 7 d had 7.4–20.6%, 14.1–36.4%, 23.7–54.2%, 24.4–50.0%, 13.4–23.5%, 13.4–28.5%, and 11.6–28.5% value increases in low-density lipoprotein, glucose, triglyceride, cholesterol, blood urea nitrogen, alanine transaminase, and aspartate amino transferase, respectively. Moreover, regarding the control rats fed for 28 d, the rats fed with GCN digest for 28 d also showed 4.2–19.7%, 12.3–35.7%, 22.8–52.8%, 10.6%–41.0%, 8.5–27.7%, 10.6–31.8%, and 8.6–24.5% value increases in the 7 indices, respectively. In addition, the rats fed with GCN digest had higher values for these indices involved in oxidative stress of the body (e.g., low-density lipoprotein, glucose, triglyceride, and cholesterol) than the control rats. This fact means GCN digest in the rats caused oxidative stress. It is thus inferred that the caseinate lactose-glycation of the Maillard-type might have adverse effects on the body.

## 4. Discussion

Despite the controversies, the Maillard reaction products, including AGEs and glycated products, are regarded to have adverse effects on cells, animals, and even human health. Our previous study had found that the GCN digest (20–500 μg/mL) in IEC-6 cells showed weaker proliferation and anti-apoptosis than CN digest; moreover, GCN digest at dose level of 500 μg/mL even displayed a toxic effect on the cells [19]. In the lipopolysaccharide-injured IEC-6 cells, it was also observed that caseinate digest had a higher protective effect than the lactose-glycated caseinate digest to promote cell growth and decrease lactate dehydrogenase release [30]. It is thus inferred that both amino acid (e.g., Lys) loss and structural change (lactose conjugation) caused decreased activity for the GCN digest. In addition, when the rats consumed a diet containing Maillard reaction products, they were observed with lower body weights [31,32]. Consistently, it was observed in this study that the rats fed with GCN digest showed lower daily gain of BW than those fed with CN digest of the same dose level, together with lower gain of the small intestine length and weight (Figure 1 and Table S1). More importantly, GCN digest at dose level of 400 mg/kg BW/d led to the lowest values for these assessed indices, indicating that the Maillard reaction products could cause an adverse effect on the rats, because higher GCN intake level meant much intake of these reaction products. The present results thus were consistent with those from the previous three studies [19,30,32], revealing an adverse effect of the Maillard-type lactose-glycation on caseinate. That is, this glycation reduced the bioactivity of GCN digest toward the rats. It is thereby suggested that a future investigation about the effect of glycated caseinate on animal models is necessary; moreover, the relationship between the digestibility of glycated caseinate and body weight gain of the animals should also be addressed.

Intestinal morphology including villus height, crypt depth, and villus/crypt ratio reflects critical digestion and absorption capacity of nutrients in the intestine, and correlates with intestinal health [33]. Intestinal villi are the major sites for nutrient absorption; consequentially, a higher villus height is associated with an increased surface area that ensures higher nutrient absorption [34]. The crypts, known as the villus factory where new epithelial cells are produced, can ensure renewal of the villus in response to normal sloughing or inflammation from the pathogens or their toxins [35]. Increases in the villus height and VH/CD ratio are directly correlated with the increased epithelial cell turnover [36,37]. However, the Maillard reaction products might be unbeneficial to the intestine; for example, dietary AGEs were observed to increase intestinal permeability and elevate inflammatory factor (TNF-α) levels, which then damaged the intestine and impacted host health adversely [38]. In addition, it was evident that the young (3-week) and adult (12-week) rats fed with the Maillard reaction products from glucose and lysine showed decreased protein digestibility, while this digestibility decrease was much clear in young rats rather than adult rats [39]. In this study, the rats fed with CN digest had higher values in both villus height and VH/CD ratio in the duodenum, jejunum, and ileum than those fed with GCN digest, implying that the conducted caseinate lactose-glycation also impaired the ability of GCN digest to the rats in term of critical intestine development.

The activities of digestive and brush-border enzymes are positively correlated with the digestive function of the digestive tract, which therefore might be responsible for the improved nutrient digestibility and growth performance [40]. In this study, both CN digest and GCN digest had an ability to increase the activities of these enzymes in the duodenum, jejunum, and ileum, but GCN digest always was less efficient than CN digest. In addition, the digestive enzyme activities for the control rats in this study were lower than those reported in a previous study [41]. The rats in this study were fasted for 6 h prior to the sacrifice; thus, it was reasonable that lower digestive enzyme activities were measured in the rats. Furthermore, it was also found that the activities of digestive enzymes such as carboxypeptidase, aminopeptidase, and α-amylase were inhibited by the Maillard reaction product from various reaction systems, while trypsin extracted from the anterior intestine also could be inhibited by the melanoidins [42,43,44]. The three studies thus provided result support to the present study. The rats fed with GCN digest therefore were measured with lower activities for the digestive and brush-border enzymes. Although rat BW gain was not correlated well with these assessed enzyme activities, it is also necessary to investigate the unrevealed relationship among GCN digest intake, intestinal morphology, and digestive enzyme activities.

In general, blood biochemical parameters are important indicators for describing the physiological and metabolic status of the animals. According to the cross-sectional analyses of the Cohort on Diabetes and Atherosclerosis Maastricht study (CODAM), dietary intake of AGEs is regarded to be associated with higher AGEs level in plasma and urine, such as Nε-(carboxymethyl)lysine, Nε-(1-carboxyethyl)lysine, and Nδ-(5-hydro-5-methyl-4-imidazolon-2-yl)-ornithine [45]. It was thus suggested that the high concentration of the glycated products could aggravate oxidative stress of the body, subsequently changing blood parameters to cause the development of age-related diseases [46]. A study on fish in stress had shown that their serum concentrations of glucose, triglyceride and total cholesterol, together with the activities of aspartate aminotransferase and alanine aminotransferase, were detected at elevated levels [47]. In this study, the detected serum levels of low-density lipoprotein, glucose, triglyceride, and cholesterol in the control rats were similar to those reported in another study [48]. However, the rats fed with GCN digest had value increases in aspartate aminotransferase and alanine aminotransferase activities as well as bilirubin content (Table 3). In theory, these indices are recognized as the exquisite markers of oxidative stress in the body [49,50]. The rats fed with GCN digest thus obtained extra oxidative stress, implying that the performed caseinate lactose-glycation of the Maillard-type endowed GCN digest with an unfavorable ability in the body. Furthermore, serum glucose content is governed by many factors including carbohydrate intake, carbohydrate digestibility, glycogenolysis, and insulin sensitivity [47,51]. The rats fed with GCN digest had inhibited α-amylase activity but higher serum glucose, and an investigation is also needed to further reveal this phenomenon.

## 5. Conclusions

Using the weaned rat model, it was evident that the lactose glycation of caseinate endowed the resultant GCN digest with less efficiency than unglycated CN digest to enhance indices such as body weight gain, small intestine weight, villus height, villus/crypt ratio, and activities of three digestive enzymes or three brush-border enzymes. In addition, GCN digest could induce extra oxidative stress in the rats by increasing the values of the related serum biochemical markers. The present results thus highlighted an adverse effect of the Maillard-type glycation on major milk proteins for their critical biofunctions in the body; that is, lactose glycation of the Maillard-type impairs the benefits of casein digest to the weaned rats in the critical body weight gain, intestinal development, and serum biochemistry. The present results thus might encourage the dairy industry to apply a suitable thermal treatment during milk processing in an effort to reduce the potential formation of Maillard reaction products at the early reaction stage.

## Figures and Tables

**Table 1:** Measured activities ^1^ of digestive enzymes of the rats in response to the intake of caseinate (CN) digest and glycated caseinate (GCN) digest.

Index	Time (d)	Control	CN Digest (mg/kg BW/d)			GCN Digest (mg/kg BW/d)		
			100	200	400	100	200	400
**Duodenum**								
Lipase	7	126.90 ± 5.46 ^b^	135.04 ± 6.52 ^bc^	156.39 ± 6.69 ^d^	204.35 ± 11.17 ^e^	127.83 ± 6.24 ^b^	136.99 ± 5.34 ^c^	115.30 ± 6.53 ^a^
	28	165.56 ± 5.86 ^b,^*	196.48 ± 9.19 ^c,^*	209.89 ± 10.72 ^d,^*	231.48 ± 10.15 ^e,^*	167.46 ± 4.13 ^b,^*	198.85 ± 8.97 ^c,^*	143.91 ± 8.57 ^a,^*
α-Amylase	7	4.67 ± 0.16 ^b^	5.56 ± 0.12 ^e^	5.88 ± 0.15 ^f^	7.21 ± 0.20 ^g^	4.89 ± 0.16 ^c^	5.29 ± 0.12 ^d^	4.13 ± 0.09 ^a^
	28	5.76 ± 0.15 ^a,^*	7.16 ± 0.20 ^c,^*	8.54 ± 0.27 ^d,^*	8.74 ± 0.13 ^d,^*	6.27 ± 0.12 ^b,^*	7.04 ± 0.24 ^c,^*	5.57 ± 0.14 ^a,^*
Trypsin	7	2.82 ± 0.03 ^b^	3.64 ± 0.09 ^c^	4.24 ± 0.10 ^e^	4.99 ± 0.06 ^f^	2.89 ± 0.04 ^b^	3.74 ± 0.03 ^d^	2.71 ± 0.08 ^a^
	28	2.94 ± 0.10 ^a,^*	4.29 ± 0.18 ^c,^*	4.76 ± 0.18 ^d,^*	5.32 ± 0.09 ^e,^*	3.23 ± 0.06 ^b,^*	4.34 ± 0.07 ^c,^*	2.81 ± 0.10 ^a,^*
**Jejunum**								
Lipase	7	104.28 ± 5.43 ^a^	109.89 ± 6.01 ^a b^	136.45 ± 6.12 ^c^	187.53 ± 6.00 ^d^	107.35 ± 5.68 ^a^	122.82 ± 9.71 ^b^	101.16 ± 6.54 ^a^
	28	148.65 ± 6.47 ^b,^*	161.49 ± 6.01 ^c,^*	177.40 ± 8.09 ^d,^*	202.05 ± 11.56 ^e,^*	154.04 ± 6.02 ^b c,^*	176.87 ± 7.37 ^d,^*	123.20 ± 4.04 ^a,^*
α-Amylase	7	4.81 ± 0.14 ^b^	6.52 ± 0.13 ^d^	6.67 ± 0.10 ^d^	7.77 ± 0.24 ^e^	5.58 ± 0.07 ^c^	6.65 ± 0.11 ^d^	4.55 ± 0.17 ^a^
	28	6.25 ± 0.13 ^b,^*	8.38 ± 0.09 ^d,^*	9.23 ± 0.09 ^e,^*	10.14 ± 0.13 ^f,^*	7.66 ± 0.22 ^c,^*	9.11 ± 0.17 ^e,^*	5.96 ± 0.10 ^a,^*
Trypsin	7	2.23 ± 0.06 ^b^	3.17 ± 0.12 ^d^	3.74 ± 0.10 ^f^	4.39 ± 0.07 ^g^	2.36 ± 0.11 ^c^	3.29 ± 0.08 ^e^	2.06 ± 0.04 ^a^
	28	2.47 ± 0.12 ^b,^*	3.78 ± 0.14 ^d,^*	4.28 ± 0.06 ^e,^*	4.72 ± 0.16 ^f,^*	2.84 ± 0.09 ^c,^*	3.72 ± 0.08 ^d,^*	2.00 ± 0.11 ^a,^*
**Ileum**								
Lipase	7	78.92 ± 4.13 ^a^	95.39 ± 4.53 ^b^	122.70 ± 9.13 ^d^	164.17 ± 9.57 ^e^	85.20 ± 5.86 ^a^	113.49 ± 8.13 ^c^	86.56 ± 4.29 ^a,b^
	28	114.66 ± 10.45 ^b,^*	126.25 ± 10.88 ^c,^*	146.10 ± 8.09 ^d,^*	184.82 ± 7.86 ^e,^*	121.71 ± 5.71 ^b,c,^*	144.29 ± 9.51 ^d,^*	95.31 ± 5.14 ^a,^*
α-Amylase	7	4.34 ± 0.09 ^b^	5.14 ± 0.14 ^d^	5.58 ± 0.18 ^e^	7.12 ± 0.21 ^f^	4.74 ± 0.09 ^c^	5.25 ± 0.08 ^d^	4.07 ± 0.15 ^a^
	28	5.55 ± 0.23 ^b,^*	6.02 ± 0.17 ^c,^*	7.94 ± 0.09 ^d,^*	8.47 ± 0.30 ^e,^*	5.86 ± 0.09 ^b,c,^*	6.07 ± 0.13 ^c,^*	5.26 ± 0.12 ^a,^*
Trypsin	7	1.82 ± 0.05 ^a b^	2.48 ± 0.08 ^c^	3.43 ± 0.05 ^e^	3.76 ± 0.05 ^f^	1.89 ± 0.02 ^b^	2.84 ± 0.05 ^d^	1.77 ± 0.04 ^a^
	28	2.25 ± 0.14 ^b,^*	3.21 ± 0.08 ^d,^*	3.70 ± 0.10 ^e,^*	3.63 ± 0.06 ^e,^*	2.26 ± 0.15 ^b,^*	3.03 ± 0.26 ^c^	1.81 ± 0.06 ^a,^*

Values are reported as means ± standard deviations (*n* = 6), while different lowercase letters after the values as the superscripts in the same row indicate that one-way ANOVA of the means differs significantly (*p* < 0.05). Paired samples *t* test is used to determine the differences of the same enzyme activity between 7 and 28 d, while the asterisk indicates data difference (*p* < 0.05). ^1^ The activities of α-amylase and trypsin are expressed as U/mg protein, while lipase is expressed as U/g protein.

**Table 2:** Measured activities ^1^ of critical brush-border enzymes of the rats in response to the intake of caseinate (CN) digest and glycated caseinate (GCN) digest.

Index	Time (d)	Control	CN Digest (mg/kg BW/d)			GCN (mg/kg BW/d)		
			100	200	400	100	200	400
**Duodenum**								
Lactase	7	9.81 ± 0.13 ^a,b^	10.44 ± 0.34 ^b,c^	12.16 ± 0.62 ^d^	12.46 ± 0.77 ^d^	10.30 ± 0.57 ^a,b,c^	10.90 ± 0.53 ^c^	9.65 ± 0.30 ^a^
	28	8.50 ± 0.39 ^b,^*	9.40 ± 0.48 ^c,^*	10.11 ± 0.22 ^d,^*	11.90 ± 0.29 ^e^	8.64 ± 0.29 ^b,^*	9.60 ± 0.33 ^c,^*	8.05 ± 0.20 ^a,^*
Sucrase	7	23.01 ± 1.36 ^b^	27.61 ± 1.37 ^c^	32.20 ± 2.38 ^d^	42.62 ± 1.48 ^e^	24.10 ± 1.62 ^b^	28.62 ± 1.86 ^c^	20.23 ± 1.52 ^a^
	28	30.20 ± 1.32 ^a,^*	37.26 ± 1.61 ^c,^*	46.47 ± 2.35 ^d,^*	52.60 ± 1.55 ^e,^*	33.42 ± 1.63 ^b,^*	39.31 ± 2.11 ^c,^*	28.22 ± 1.16 ^a,^*
Alkaline phosphatase	7	141.93 ± 10.06 ^a b^	173.31 ± 9.58 ^c^	223.54 ± 10.77 ^d^	232.87 ± 8.43 ^d^	151.06 ± 7.51 ^b^	173.54 ± 13.70 ^c^	135.44 ± 7.00 ^a^
	28	198.35 ± 11.89 ^b,^*	224.68 ± 15.16 ^c,^*	242.57 ± 17.74 ^d^	264.94 ± 11.74 ^e,^*	204.08 ± 11.41 ^b,^*	227.34 ± 6.55 ^c d,^*	176.80 ± 5.60 ^a,^*
**Jejunum**								
Lactase	7	8.75 ± 0.16 ^a^	9.76 ± 0.11 ^b^	11.14 ± 0.24 ^c^	11.43 ± 0.32 ^c^	9.55 ± 0.43 ^b^	9.98 ± 0.79 ^b^	8.27 ± 0.46 ^a^
	28	7.55 ± 0.23 ^a,^*	8.97 ± 0.25 ^b,^*	9.13 ± 0.39 ^b,^*	10.70 ± 0.22 ^c,^*	7.82 ± 0.37 ^a,^*	9.16 ± 0.32 ^b,^*	7.78 ± 0.22 ^a,^*
Sucrase	7	29.71 ± 1.25 ^a^	35.58 ± 1.28 ^b^	44.07 ± 2.09 ^c^	50.30 ± 2.43 ^d^	30.43 ± 1.39 ^a^	37.9 ± 2.42 ^b^	27.97 ± 1.57 ^a^
	28	38.45 ± 1.38 ^b,^*	54.45 ± 2.36 ^d,^*	60.46 ± 3.13 ^e,^*	62.35 ± 2.31 ^e,^*	41.25 ± 1.74 ^c,^*	54.88 ± 1.77 ^d,^*	33.27 ± 1.65 ^a,^*
Alkaline phosphatase	7	113.37 ± 8.72 ^a,b^	133.86 ± 6.88 ^c^	173.89 ± 6.61 ^e^	198.70 ± 10.85 ^f^	121.25 ± 7.49 ^b^	147.22 ± 8.04 ^d^	107.90 ± 6.91 ^a^
	28	161.49 ± 6.70 ^a,^*	172.37 ± 6.16 ^b,^*	226.82 ± 4.87 ^d,^*	232.30 ± 8.26 ^d,^*	171.04 ± 7.18 ^b,^*	188.85 ± 7.76 ^b,c,^*	158.67 ± 6.21 ^a,^*
**Ileum**								
Lactase	7	7.84 ± 0.04 ^a^	8.80 ± 0.12 ^b^	9.05 ± 0.51 b ^c^	9.51 ± 0.54 ^c^	8.57 ± 0.42 ^b^	8.84 ± 0.58 ^b^	7.64 ± 0.40 ^a^
	28	6.93 ± 0.15 ^b,^*	8.03 ± 0.24 ^c,^*	8.30 ± 0.32 ^c,^*	9.01 ± 0.34 ^d,^*	7.00 ± 0.45 ^b,^*	8.05 ± 0.46 ^c,^*	6.34 ± 0.15 ^a,^*
Sucrase	7	16.50 ± 0.65 ^a^	22.41 ± 1.03 ^c^	26.66 ± 1.74 ^d^	37.27 ± 0.72 ^e^	18.66 ± 1.62 ^b^	23.62 ± 1.14 ^c^	16.50 ± 0.47 ^a^
	28	23.65 ± 1.31 ^a,^*	33.41 ± 1.70 ^c,^*	38.14 ± 0.83 ^d,e,^*	41.21 ± 2.05 ^e,^*	29.81 ± 1.72 ^b,^*	36.39 ± 2.03 ^d,^*	21.60 ± 1.40 ^a,^*
Alkaline phosphatase	7	99.95 ± 5.47 ^a,b^	120.5 ± 7.13 ^c^	147.63 ± 5.30 ^e^	156.78 ± 11.72 ^e^	108.19 ± 7.81 ^b^	131.06 ± 7.59 ^d^	94.38 ± 3.15 ^a^
	28	145.16 ± 9.92 ^a,b,^*	152.85 ± 5.77 ^b,^*	176.78 ± 5.46 ^c,^*	201.60 ± 6.87 ^d,^*	151.29 ± 5.35 ^b,^*	152.18 ± 5.90 ^b,^*	139.56 ± 7.58 ^a,^*

Values are reported as means ± standard deviations (*n* = 6), while different lowercase letters after the values as the superscripts in the same row indicate that one-way ANOVA of the means differs significantly (*p* < 0.05). Paired samples *t* test is used to determine the differences of the same enzyme activity between 7 and 28 d, while the asterisk indicates data difference (*p* < 0.05). ^1^ Both lactase and sucrase activities are expressed as nmol glucose/(mg protein min), whereas alkaline phosphatase activity is expressed as U/mg protein.

**Table 3:** Changes of serum biochemistry (mmol/L) of the rats in response to the intake of caseinate (CN) digest and glycated caseinate (GCN) digest.

Index	Time (d)	Control	CN Digest (mg/kg BW/d)			GCN Digest (mg/kg BW/d)		
			100	200	400	100	200	400
Low-density lipoprotein	7	0.68 ± 0.04 ^a^	0.70 ± 0.04 ^a,b^	0.72 ± 0.02 ^a,b^	0.72 ± 0.03 ^a,b^	0.73 ± 0.02 ^b^	0.79 ± 0.02 ^c^	0.82 ± 0.04 ^c^
	28	0.71 ± 0.03 ^a^	0.73 ± 0.05 ^a,b^	0.74 ± 0.03 ^a,b,^*	0.75 ± 0.06 ^a,b^	0.74 ± 0.04 ^a,b^	0.80 ± 0.05 ^b,c^	0.85 ± 0.07 ^c^
Glucose	7	3.98 ± 0.11 ^a^	4.05 ± 0.13 ^a,b^	4.17 ± 0.07 ^b,c^	4.23 ± 0.13 ^c^	4.54 ± 0.11 ^d^	5.04 ± 0.15 ^e^	5.43 ± 0.14 ^f^
	28	4.06 ± 0.09 ^a,^*	4.17 ± 0.12 ^a,b,^*	4.33 ± 0.14 ^b,c^	4.41 ± 0.11 ^c,^*	4.56 ± 0.12 ^c^	5.21 ± 0.17 ^d,^*	5.51 ± 0.15 ^e^
Triglyceride	7	1.18 ± 0.05 ^a^	1.20 ± 0.06 ^a^	1.27 ± 0.05 ^a,b^	1.32 ± 0.05 ^b^	1.46 ± 0.09 ^c^	1.53 ± 0.08 ^c^	1.82 ± 0.06 ^d^
	28	1.23 ± 0.04 ^a,^*	1.24 ± 0.04 ^a,^*	1.28 ± 0.04 ^a,b^	1.35 ± 0.05 ^b,^*	1.51 ± 0.05 ^c,^*	1.56 ± 0.10 ^c^	1.88 ± 0.06 ^d^
Cholesterol	7	1.72 ± 0.04 ^a^	1.77 ± 0.04 ^a^	1.89 ± 0.08 ^b^	2.02 ± 0.13 ^c^	2.14 ± 0.07 ^d^	2.38 ± 0.08 ^e^	2.58 ± 0.05 ^f^
	28	1.88 ± 0.05 ^a,^*	1.91 ± 0.06 ^a,^*	2.02 ± 0.06 ^b,^*	2.13 ± 0.08 ^c,^*	2.08 ± 0.08 ^b,c,^*	2.40 ± 0.09 ^d^	2.65 ± 0.11 ^e^
Blood urea nitrogen	7	5.54 ± 0.08 ^a^	5.66 ± 0.19 ^a^	5.76 ± 0.19 ^a,b^	5.95 ± 0.15 ^b^	6.28 ± 0.24 ^c^	6.62 ± 0.23 ^d^	6.84 ± 0.16 ^d^
	28	6.13 ± 0.33 ^a,^*	6.36 ± 0.17 ^a,b,^*	6.56 ± 0.16 ^b,c,^*	6.76 ± 0.17 ^c,^*	6.65 ± 0.18 ^b,c,^*	7.49 ± 0.24 ^d,^*	7.83 ± 0.21 ^e,^*
Alanine transaminase	7	26.86 ± 1.29 ^a^	27.56 ± 1.11 ^a^	28.71 ± 1.72 ^a,b^	29.60 ± 1.53 ^b^	30.47 ± 1.27 ^b,c^	31.97 ± 1.45 ^c^	34.51 ± 1.84 ^d^
	28	28.34 ± 2.15 ^a^	29.48 ± 1.92 ^a,b^	30.53 ± 1.53 ^a,b,^*	31.67 ± 1.89 ^b,c,^*	31.33 ± 2.78 ^a,b^	34.55 ± 2.95 ^c,d,^*	37.36 ± 2.43 ^d,^*
Aspartate amino transferase	7	172.28 ± 4.29 ^a^	175.61 ± 6.67 ^a^	179.37 ± 5.58 ^a^	187.57 ± 8.84 ^b^	192.27 ± 6.47 ^b^	209.74 ± 5.47 ^c^	221.34 ± 3.24 ^d^
	28	180.95 ± 5.28 ^a,^*	183.77 ± 5.22 ^a,b,^*	185.31 ± 4.69 ^a,b,^*	189.22 ± 5.57 ^b^	196.48 ± 2.84 ^b^	209.66 ± 3.36 ^c^	225.29 ± 6.76 ^d^

Values are reported as means ± standard deviations (*n* = 6), while different lowercase letters after the values as the superscripts in the same row indicate that one-way ANOVA of the means differs significantly (*p* < 0.05). Paired samples *t* test is used to determine the differences of the same index between 7 and 28 d, while the asterisk indicates data difference (*p* < 0.05).

## Data Availability

All data are contained within the article and Supplementary File.

## Supplementary Materials

The following are available online at https://www.mdpi.com/article/10.3390/foods10092104/s1, Table S1: Several growth performance indices of the rats in response to the intake of caseinate (CN) digest and glycated caseinate (GCN) digest, Table S2: Small intestinal morphology of the rats in different groups in response to the intake of caseinate (CN) digest and glycated caseinate (GCN) digest, Table S3: Several hematological parameters for serum biochemistry of the rats in response to the intake of caseinate (CN) digest and glycated caseinate (GCN) digest.
