# Supplementary material for: Lactose Glycation of the Maillard-Type Impairs the Benefits of Caseinate Digest to the Weaned Rats for Intestinal Morphology and Serum Biochemistry
Source: Foods. 2021 Sep 6;10(9):2104. doi: 10.3390/foods10092104 (PMC8468520; doi:10.3390/foods10092104)
Supplement: Supplementary file 1 [file foods-10-02104-s001.zip › foods-1320535-supplementary.pdf]

foods

MDPI

Supplementary Materials

Table S1. Several growth performance indices of the rats in response to the intake of caseinate (CN) digest and glycated caseinate (GCN) digest.

Index	Control	CN digest (mg/kg BW/d)			GCN digest (mg/kg BW/d)		
		100	200	400	100	200	400
Initial body weight (g)	55.27 ± 0.41^{a}	55.15 ± 0.34^{a}	55.40 ± 0.37^{a}	55.23 ± 0.32^{a}	55.43 ± 0.40^{a}	55.50 ± 0.28^{a}	55.34 ± 0.32^{a}
Body weight at day 7 (g)	110.68 ± 1.60^{b}	114.81 ± 2.20^{c}	116.40 ± 1.94^{cd}	116.70 ± 1.19^{d}	111.85 ± 1.85^{b}	114.57 ± 1.33^{c}	107.37 ± 1.28^{a}
Final body weight (g)	279.73 ± 7.79^{a}	283.63 ± 8.21^{a}	285.67 ± 11.68^{a}	286.50 ± 12.06^{a}	281.10 ± 9.57^{a}	283.67 ± 4.76^{a}	274.63 ± 7.13^{a}
Total weight gain at 0-7 d (g/d)	55.41 ± 1.63^{b}	59.66 ± 2.04^{cd}	60.99 ± 1.60^{cd}	61.47 ± 1.28^{d}	56.41 ± 1.68^{b}	59.06 ± 1.53^{c}	52.03 ± 1.54^{a}
Total weight gain at 0-28 d (g/d)	224.46 ± 7.43^{a}	228.48 ± 8.21^{a}	230.26 ± 11.33^{a}	231.27 ± 12.05^{a}	225.67 ± 9.65^{a}	228.16 ± 4.49^{a}	219.29 ± 7.18^{a}
Initial weight of small intestine (g)	2.53 ± 0.30	2.53 ± 0.30	2.53 ± 0.30	2.53 ± 0.30	2.53 ± 0.30	2.53 ± 0.30	2.53 ± 0.30
Weight of small intestine at 7 d (g)	3.87 ± 0.08^{bc}	3.95 ± 0.03^{c}	4.13 ± 0.12^{d}	4.33 ± 0.08^{e}	3.84 ± 0.07^{b}	3.96 ± 0.08^{c}	3.66 ± 0.06^{a}
Weight of small intestine at 28 d (g)	6.23 ± 0.17^{a}	6.81 ± 0.07^{c}	6.85 ± 0.10^{c}	7.06 ± 0.13^{d}	6.53 ± 0.10^{b}	6.84 ± 0.11^{c}	6.16 ± 0.17^{a}
Total gain of small intestine weight at 0-7 d (g/d)	1.34 ± 0.08^{bc}	1.42 ± 0.03^{c}	1.61 ± 0.12^{d}	1.80 ± 0.08^{e}	1.31 ± 0.07^{b}	1.43 ± 0.08^{c}	1.14 ± 0.06^{a}
Total gain of small intestine weight at the 0-28 d (g/d)	3.70 ± 0.17^{a}	4.28 ± 0.07^{c}	4.32 ± 0.10^{c}	4.53 ± 0.13^{d}	4.01 ± 0.10^{b}	4.31 ± 0.11^{c}	3.63 ± 0.17^{a}
Initial length of small intestine (cm)	64.61 ± 1.50	64.61 ± 1.50	64.61 ± 1.50	64.61 ± 1.50	64.61 ± 1.50	64.61 ± 1.50	64.61 ± 1.50
length of small intestine at 7 d (cm)	71.07 ± 1.24^{ab}	72.51 ± 0.94^{cd}	73.62 ± 0.97^{de}	74.92 ± 1.37^{e}	71.70 ± 0.61^{bc}	72.65 ± 1.16^{cd}	69.82 ± 0.75^{a}
length of small intestine at 28 d (cm)	92.43 ± 1.55^{b}	95.58 ± 1.02^{c}	97.13 ± 1.42^{c}	99.83 ± 1.16^{d}	91.20 ± 1.36^{b}	91.90 ± 2.26^{b}	88.53 ± 2.66^{a}
Total length gain of small intestine at 0-7 d (cm)	6.46 ± 1.24^{ab}	7.90 ± 0.94^{cd}	9.01 ± 0.97^{de}	10.31 ± 1.37^{e}	7.09 ± 0.61^{bc}	8.04 ± 1.16^{cd}	5.21 ± 0.57^{a}
Total length gain of small intestine at 0-28 d (cm)	27.82 ± 1.55^{b}	30.97 ± 1.02^{c}	32.52 ± 1.42^{c}	35.22 ± 1.16^{d}	26.59 ± 1.36^{b}	27.29 ± 2.26^{b}	23.92 ± 2.66^{a}

Values are reported as means ± standard deviations (n = 6); different lowercase letters after the values as the superscripts in the same row indicate that one-way ANOVA of the means differs significantly ($p < 0.05$).

Table S2. Small intestinal morphology of the rats in different groups in response to the intake of caseinate (CN) digest and glycated caseinate (GCN) digest.

Index	Time (d)	Control	CN digest (mg/kg BW/d)			GCN digest (mg/kg BW/d)		
			100	200	400	100	200	400
**Duodenum**								
VH (μm)	7	352.30 ± 11.33^{b}	386.10 ± 6.55^{d}	401.30 ± 9.91^{e}	413.63 ± 7.34^{f}	366.87 ± 12.85^{c}	381.93 ± 7.11^{d}	332.9 ± 7.29^{a}
	28	373.18 ± 9.61^{b}	400.35 ± 11.80^{c}	414.32 ± 5.80^{cd}	426.10 ± 6.65^{e}	378.03 ± 4.73^{b}	401.81 ± 5.12^{c}	337.20 ± 4.65^{a}
CD (μm)	7	139.80 ± 8.02^{cd}	132.23 ± 4.07^{ab}	128.67 ± 4.24^{ab}	127.30 ± 3.26^{a}	134.30 ± 2.04^{bc}	130.77 ± 2.50^{ab}	142.47 ± 3.65^{d}
	28	161.87 ± 4.66^{e}	146.26 ± 5.47^{c}	138.85 ± 5.10^{ab}	133.42 ± 6.72^{a}	155.41 ± 3.50^{d}	141.21 ± 1.29^{bc}	155.08 ± 5.12^{d}
VH/CD	7	2.52 ± 0.10^{b}	2.92 ± 0.10^{d}	3.12 ± 0.11^{e}	3.25 ± 0.07^{f}	2.73 ± 0.06^{c}	2.92 ± 0.04^{d}	2.34 ± 0.04^{a}
	28	2.31 ± 0.09^{b}	2.74 ± 0.08^{d}	2.99 ± 0.10^{e}	3.20 ± 0.16^{f}	2.43 ± 0.03^{c}	2.85 ± 0.06^{d}	2.18 ± 0.04^{a}
**Jejunum**								
VH (μm)	7	331.07 ± 8.46^{b}	371.87 ± 5.61^{d}	390.10 ± 8.53^{e}	395.57 ± 6.76^{e}	360.53 ± 11.32^{c}	371.43 ± 6.72^{d}	316.20 ± 8.84^{a}
	28	363.94 ± 11.28^{b}	386.01 ± 5.21^{c}	398.49 ± 7.33^{d}	410.40 ± 12.51^{e}	369.9 ± 4.48^{b}	394.10 ± 8.87^{cd}	322.19 ± 4.35^{a}
CD (μm)	7	135.20 ± 5.09^{bc}	134.47 ± 2.18^{b}	128.77 ± 5.42^{a}	123.80 ± 4.64^{a}	136.43 ± 3.08^{b}	128.07 ± 2.68^{a}	139.40 ± 2.59^{b}
	28	157.61 ± 2.89^{c}	143.10 ± 9.96^{b}	134.11 ± 4.99^{ab}	131.29 ± 8.46^{a}	157.5 ± 4.66^{c}	140.90 ± 9.53^{b}	154.71 ± 5.03^{c}
VH/CD	7	2.45 ± 0.09^{b}	2.77 ± 0.03^{cd}	3.03 ± 0.14^{e}	3.20 ± 0.16^{f}	2.64 ± 0.12^{c}	2.90 ± 0.07^{de}	2.27 ± 0.10^{a}
	28	2.31 ± 0.06^{b}	2.71 ± 0.20^{bc}	2.97 ± 0.10^{de}	3.13 ± 0.14^{e}	2.35 ± 0.07^{b}	2.81 ± 0.20^{cd}	2.08 ± 0.07^{a}
**Ileum**								
VH (μm)	7	239.17 ± 10.41^{b}	263.9 ± 6.97^{c}	268.03 ± 10.54^{c}	273.73 ± 14.53^{c}	242.77 ± 10.01^{b}	249.8 ± 5.08^{b}	219.8 ± 5.93^{a}
	28	260.93 ± 12.25^{ab}	277.26 ± 9.12^{cd}	288.64 ± 6.70^{d}	284.14 ± 9.60^{d}	266.58 ± 4.89^{abc}	271.45 ± 7.66^{bc}	258.35 ± 13.06^{a}
CD (μm)	7	104.43 ± 5.58^{a}	103.27 ± 4.10^{a}	102.97 ± 3.79^{a}	99.5 ± 5.21^{a}	97.27 ± 2.95^{a}	98.93 ± 7.82^{a}	100.90 ± 3.50^{a}
	28	129.52 ± 5.80^{bc}	124.66 ± 4.55^{b}	116.52 ± 3.38^{a}	113.84 ± 6.70^{a}	125.96 ± 4.08^{b}	115.58 ± 2.90^{a}	133.52 ± 4.83^{c}
VH/CD	7	2.30 ± 0.17^{a}	2.56 ± 0.17^{bc}	2.61 ± 0.13^{bc}	2.76 ± 0.23^{c}	2.50 ± 0.05^{b}	2.54 ± 0.19^{b}	2.18 ± 0.03^{a}
	28	2.02 ± 0.13^{ab}	2.23 ± 0.10^{cd}	2.48 ± 0.09^{ef}	2.50 ± 0.15^{f}	2.12 ± 0.04^{bc}	2.35 ± 0.11^{de}	1.94 ± 0.12^{a}

VH = Villus height; CD = Crypt depth; VH/CD = Villus height to crypt depth ratio. Values are reported as means ± standard deviations; different lowercase letters after the values as the superscripts in the same row indicate that one-way ANOVA of the means differs significantly ($p < 0.05$).

Table S3. Several hematological parameters for serum biochemistry of the rats in response to the intake of caseinate (CN) digest and glycated caseinate (GCN) digest.

Index	Time (d)	Control	CN digest (mg/kg BW/d)			GCN digest (mg/kg BW/d)		
			100	200	400	100	200	400
Total protein (g/L)	7	42.21 ± 1.55[b]	44.39 ± 1.17[cd]	45.47 ± 1.34[de]	47.29 ± 1.36[bc]	42.41 ± 1.36[bc]	43.17 ± 1.36[bc]	39.72 ± 2.16[a]
	28	47.44 ± 2.59[b]	48.70 ± 2.84[b]	49.84 ± 2.44[bc]	52.49 ± 3.28[c]	46.55 ± 2.75[b]	47.95 ± 2.75[b]	41.27 ± 2.27[a]
High-density lipoprotein	7	1.18 ± 0.05[a]	1.32 ± 0.06[abc]	1.45 ± 0.07[cd]	1.57 ± 0.07[d]	1.26 ± 0.10[ab]	1.35 ± 0.08[bc]	1.17 ± 0.05[a]
	28	1.21 ± 0.06[a]	1.44 ± 0.06[c]	1.57 ± 0.07[d]	1.67 ± 0.05[e]	1.35 ± 0.06[b]	1.48 ± 0.07[c]	1.20 ± 0.06[a]
Albumin (g/L)	7	30.66 ± 1.68[ab]	32.19 ± 1.21[ab]	35.35 ± 1.61[c]	35.27 ± 1.06[c]	31.28 ± 1.60[ab]	32.54 ± 1.79[b]	30.20 ± 1.67[a]
	28	34.31 ± 2.41[ab]	35.22 ± 2.23[a]	36.72 ± 1.80[b]	37.60 ± 2.22[b]	34.70 ± 2.56[ab]	37.03 ± 2.93[b]	32.46 ± 1.72[a]
Globulin (g/L)	7	9.67 ± 0.43[a]	11.83 ± 0.70[cd]	12.55 ± 0.70[de]	13.14 ± 1.05[e]	10.79 ± 0.49[b]	11.49 ± 0.56[bc]	9.36 ± 0.26[a]
	28	10.71 ± 0.26[b]	12.13 ± 0.84[c]	13.77 ± 0.63[d]	15.61 ± 0.85[e]	11.25 ± 0.52[b]	12.46 ± 0.53[c]	9.75 ± 0.57[a]
Creatinine (μmol/L)	7	32.98 ± 2.01[a]	33.36 ± 1.61[a]	34.33 ± 1.73[a]	34.84 ± 2.10[a]	38.00 ± 1.81[b]	39.38 ± 2.23[b]	41.83 ± 1.72[c]
	28	34.78 ± 2.18[a]	35.08 ± 2.41[a]	36.76 ± 1.38[a]	37.11 ± 1.08[a]	37.20 ± 1.57[a]	42.32 ± 1.57[b]	47.32 ± 2.41[c]
Creatinine (μmol/L)	7	108.37 ± 5.13[a]	110.69 ± 5.34[ab]	115.60 ± 4.29[bc]	117.34 ± 4.16[c]	119.12 ± 2.64[c]	124.70 ± 3.28[d]	130.20 ± 4.33[e]
	28	109.15 ± 5.73[a]	111.81 ± 4.30[ab]	115.51 ± 4.52[ab]	118.78 ± 4.55[bc]	116.59 ± 5.65[b]	125.43 ± 6.38[c]	132.89 ± 5.05[d]
Total bilirubin (μmol/L)	7	0.69 ± 0.05[a]	0.73 ± 0.06[ab]	0.75 ± 0.05[b]	0.77 ± 0.04[b]	0.76 ± 0.03[b]	0.85 ± 0.04[c]	0.93 ± 0.04[d]
	28	0.76 ± 0.05[a]	0.80 ± 0.04[ab]	0.81 ± 0.04[ab]	0.83 ± 0.04[b]	0.93 ± 0.04[c]	1.03 ± 0.03[d]	1.11 ± 0.05[e]
Alkaline phosphatase (IU/L)	7	215.00 ± 4.33[a]	217.03 ± 3.67[a]	219.12 ± 4.89[ab]	221.29 ± 2.99[ab]	226.36 ± 7.22[b]	247.26 ± 8.59[c]	277.02 ± 10.05[d]
	28	218.10 ± 8.30[a]	220.25 ± 6.06[a]	228.08 ± 6.71[b]	231.13 ± 4.45[b]	229.21 ± 6.81[b]	249.30 ± 5.06[c]	281.25 ± 4.02[d]

Values are reported as means ± standard deviations (n = 6); different lowercase letters after the values as the superscripts in the same row indicate that one-way ANOVA of the means differs significantly ($p < 0.05$).
